# Identification and Characterization of ten *Escherichia coli* Strains Encoding Novel Shiga Toxin 2 Subtypes, Stx2n as Well as Stx2j, Stx2m, and Stx2o, in the United States

**DOI:** 10.3390/microorganisms11102561

**Published:** 2023-10-14

**Authors:** Rebecca L. Lindsey, Arjun Prasad, Michael Feldgarden, Narjol Gonzalez-Escalona, Curtis Kapsak, William Klimke, Angela Melton-Celsa, Peyton Smith, Alexandre Souvorov, Jenny Truong, Flemming Scheutz

**Affiliations:** 1 Enteric Diseases Laboratory Branch, Centers for Disease Control and Prevention, Atlanta, GA 30333, USA; kapsakcj@gmail.com (C.K.); koj1@cdc.gov (P.S.); 2 National Center for Biotechnology Information, National Library of Medicine, National Institutes of Health, Bethesda, MD 20894, USA; aprasad@mail.nih.gov (A.P.); michael.feldgarden@nih.gov (M.F.); klimke@ncbi.nlm.nih.gov (W.K.); souvorov@ncbi.nlm.nih.gov (A.S.); 3 Center for Food Safety and Applied Nutrition, Food and Drug Administration, College Park, MD 20740, USA; narjol.gonzalez-escalona@fda.hhs.gov; 4 Department of Microbiology and Immunology, School of Medicine, Uniformed Services University of the Health Sciences, Bethesda, MD 20184, USA; angela.melton-celsa@usuhs.edu; 5 Oak Ridge Institute for Science and Education, Oak Ridge, TN 37830, USA; odv3@cdc.gov; 6 The International Escherichia and Klebsiella Centre, Statens Serum Institut, 2300 Copenhagen, Denmark; fsc@ssi.dk

**Keywords:** Shiga toxin-producing *Escherichia coli*, Shiga toxin subtype, Stx2n, genome sequence

## Abstract

The sharing of genome sequences in online data repositories allows for large scale analyses of specific genes or gene families. This can result in the detection of novel gene subtypes as well as the development of improved detection methods. Here, we used publicly available WGS data to detect a novel Stx subtype, Stx2n in two clinical *E. coli* strains isolated in the USA. During this process, additional Stx2 subtypes were detected; six Stx2j, one Stx2m strain, and one Stx2o, were all analyzed for variability from the originally described subtypes. Complete genome sequences were assembled from short- or long-read sequencing and analyzed for serotype, and ST types. The WGS data from Stx2n- and Stx2o-producing STEC strains were further analyzed for virulence genes pro-phage analysis and phage insertion sites. Nucleotide and amino acid maximum parsimony trees showed expected clustering of the previously described subtypes and a clear separation of the novel Stx2n subtype. WGS data were used to design OMNI PCR primers for the detection of all known *stx*1 (283 bp amplicon), *stx*2 (400 bp amplicon), intimin encoded by *eae* (221 bp amplicon), and *stx*2f (438 bp amplicon) subtypes. These primers were tested in three different laboratories, using standard reference strains. An analysis of the complete genome sequence showed variability in serogroup, virulence genes, and ST type, and Stx2 pro-phages showed variability in size, gene composition, and phage insertion sites. The strains with Stx2j, Stx2m, Stx2n, and Stx2o showed toxicity to Vero cells. Stx2j carrying strain, 2012C-4221, was induced when grown with sub-inhibitory concentrations of ciprofloxacin, and toxicity was detected. Taken together, these data highlight the need to reinforce genomic surveillance to identify the emergence of potential new Stx2 or Stx1 variants. The importance of this surveillance has a paramount impact on public health. Per our description in this study, we suggest that 2017C-4317 be designated as the Stx2n type-strain.

## 1. Introduction

Shiga toxin (Stx) is the defining virulence factor in Shiga Toxin-Producing *Escherichia coli* (STEC), which can cause gastrointestinal illness with possible life-threatening complications in humans. Two major types of Stx, Stx1 and Stx2, are further divided into subtypes, Stx1 (a, c, d) and 14 Stx2 (a-m, o). Recently, Stx2 (j, m, and o) have been described [1,2].

A 2020 study from the EU found that O157 STEC was only isolated in 20.6% of the confirmed cases of human STEC infections; the remaining 79.4% of confirmed cases were associated with non-O157 STEC [3]. Therefore, methods for the detection of toxin genes instead of or in addition to serogroup detection are important diagnostic tools for STEC infection.

In 2012, a standardized Stx nomenclature was established for Stx1/Stx2 and associated subtypes, which included Stx2a-Stx2g [4]. In the last ten years, additional Stx2 subtypes, Stx2h-Stx2m, have been described and named following the standardized Stx nomenclature: Stx2h [5], Stx2i [6], Stx2j [1], Stx2k [7], Stx2l [8], Stx2m [2], and Stx2o [1].

On 2 February 2020, as part of the evaluation of the AMRFinderPlus tool [9] for the detection of *stx* variants in the Pathogen Detection system, we found 116 (0.2%) genomes among over 60,000 *E. coli* and *Shigella* genomes screened with *stx*_2_ B subunit sequences that fell just below the cutoffs for *stx*_2_ subtypes a through g. Several of the new variants identified were in the process of being characterized and published by other groups (e.g., Stx2j, Stx2m, and Stx2o) [1,2]. Among these 116 genomes, 2 were positive for a novel *stx*_2_ subtype, provisionally designated *stx*_2n_. Ten isolates, six Stx2j, one Stx2m, two Stx2n, and one Stx2o, were selected for further analysis and characterization for this study.

## 2. Materials and Methods

### 2.1. Detection of Novel Stx2 Subtypes

Over 60,000 *Escherichia coli* and *Shigella* isolates with short-read data included in the National Center for Biotechnology Information (NCBI) Pathogen Detection System (https://www.ncbi.nlm.nih.gov/pathogens, accessed on 11 October 2023) as of 2 February 2020 were screened by both de novo assembly using SKESA [10] and targeted assembly using SAUTE with characterized *stx* sequences as targets [11]. Resulting assemblies were analyzed with AMRFinderPlus which includes curated Hidden Markov models (HMMs) that can identify novel divergent *stx* genes [9]. Novel strains from the USA were selected for further analysis.

### 2.2. Collection of STEC Strains

The standard operating procedure (SOP) for PulseNet USA, the molecular surveillance network for foodborne disease in the United States [12], includes the uploading of raw sequence reads to NCBI, where the novel *stx* subtypes were detected. Limited metadata are available within the NCBI BioSample records for these clinical isolates due to laws that prevent the sharing of personally identifiable information (PII) [13].

### 2.3. Illumina and Oxford Nanopore Sequencing and Assembly

DNA was extracted from bacterial cells using the Promega Wizard kit (Promega Wizard Genomic DNA Purification Kit, Promega Corporation, Madison, WI, USA), and wide-bore pipette tips and minimal handling were used to produce high-molecular-weight DNA. A single DNA extract was used for all sequencing methods. Illumina MiSeq libraries were prepared with the DNA Prep Library kit (Illumina, Inc., San Diego, CA, USA), using modified bead ratios for optimal fragment size, following the PulseNet standard operating procedure (SOP) PNL35 (https://www.cdc.gov/pulsenet/pathogens/wgs.html, accessed on 11 October 2023) and sequenced to a minimum of 40X coverage [14]. Nanopore MinION libraries were prepared with the Rapid Barcoding kit according to the manufacturer’s protocol, without size selection or normalization, and sequenced for 72 h on the R9.4.1 flow cell (Oxford Nanopore Technologies, Oxford, UK).

Illumina raw reads were analyzed with a PulseNet customized version of BioNumerics (Applied Maths, Sint-Martens-Latem, Belgium), a commercial off-the-shelf data analysis and management software, to assemble (SPAdes v2.2) and then analyze the WGS [12].

The complete hybrid genome assemblies comprising the chromosome and plasmid(s) for the Stx2n and Stx2o isolates were obtained by de novo assembly using both Illumina and Nanopore data with Unicycler v0.4.8 [15]. Additionally, secondary genome assemblies were obtained by de novo assembly using Nanopore data only, with Flye v2.8 [16]. The hybrid and Nanopore-only assemblies for each isolate were aligned with Mauve v2.4.0 [17] to look for any disagreement in synteny, size, or completeness.

Since both genome assemblies (hybrid and Nanopore-only) for these three isolates agreed regarding all these requirements, the hybrid assembly was determined to be the final assembly (i.e., complete genome). Unicycler assembled the chromosome and plasmids as circular closed and oriented the chromosome to start at the *dnaA* gene. The genomes were annotated using the NCBI Prokaryotic Genome Annotation Pipeline (PGAP v5.0, http://www.ncbi.nlm.nih.gov/genome/annotation_prok, accessed on 11 October 2023) [18].

### 2.4. WGS-Based Characterization

The serotype and virulence gene content of the Stx2j, Stx2m, Stx2n and Stx2o assemblies were identified using the Center for Genomic Epidemiology (http://www.genomicepidemiology.org, accessed on 11 October 2023) web-based Serotype Finder 2.0.2 and Virulence Finder 1.5 tools [19,20,21].

Multi-Locus Sequence Types were obtained by using Torsten Seemann’s in silico *E. coli* MLST approach, with command line software mlst v2.23.0 (GitHub-tseemann/mlst: :id: Scan contig files against PubMLST typing schemes). Seven housekeeping genes (*adk*, *fumC*, *gyrB*, *icd*, *mdh*, *purA*, and *recA*), described previously for *E. coli* [22], were used for MLST analysis and to assign numbers for alleles and sequence type (ST).

### 2.5. Stx Subtyping

The Stx subtypes of the selected STEC isolates were determined by ABRicate version 0.8.10 (https://github.com/tseemann/abricate, accessed on 1 March 2020) with the default parameters. Briefly, a stx subtyping database was created with ABRicate by including representative nucleotide sequences of all identified Stx1 and Stx2 subtypes.

The assemblies were then searched against the *stx* subtyping database. For the *stx* genes that yield an identity below 96% with the nearest known stx subtype, the full nucleotide sequences were extracted and compared to the GenBank database with the NCBI Blast tool.

The representative nucleotide sequences of all the *stx*_2_ subtypes and variants (*stx2a-stx2m*, *stx2o*) described previously were downloaded from GenBank (Stx2h [5], Stx2i [6], Stx2j [1], Stx2k [7], Stx2l [8], Stx2m [2], Stx2o [1]). The amino acid sequences for the combined A and B subunits of Stx2 holotoxin were translated from the open reading frames. The full amino acid and nucleotide sequences were aligned to calculate the genetic distances between *stx*_2_/Stx2 sequences Evolutionary unrooted trees were created from maximum parsimony cluster analysis using 100 bootstrap resamples. Also, the amino acid sequences were analyzed for sequence motifs that support the phylogenetic analyses using BioNumerics version 7.6 (Applied Maths, Ghent, Belgium), as previously described Scheutz, F. et al. [4].

### 2.6. Cytotoxicity, Ciprofloxacin (Cip) Induction, and Activation Assays

The level of cytotoxicity from culture supernatant fractions or cell fractions were determined on Vero cells as previously described [23]. Sub-lethal concentrations of ciprofloxacin (5 ng/mL) were added to some cultures for evaluation of induction. The toxins were tested for activation by incubation of the supernatant fractions with mouse intestinal mucus or a buffer control for 1–2 h then determining the toxicity on Vero cells as described previously [24].

### 2.7. Polymerase Chain Reaction (PCR) Primers to Detect All Described stx1 and stx2 Subtypes

Primers for this study were redesigned and tested independently in three laboratories for the detection of all 18 Stx1/Stx2 subtypes (Table 1). PCR was performed for each target in a total volume of 20 µL with 10 µL HotStarTaq Master Mix Kit (Qiagen, Venlo, The Netherlands), 5 µL of primer mix (20 µM each primer), and 5 µL supernatant of boiled lysate. The thermocycler conditions were 95 °C for 15 min, followed by 35 cycles of 94 °C for 50 s, 62 °C for 40 s, and 72 °C for 50 s, ending with 72 °C for 3 min. PCR amplicons were stored at 4 °C. Amplicons were separated on a 2% agarose gel stained with GelRed for a total of 30 min at 100 volts.

### 2.8. Stx2n and Stx2o Pro-Phages Annotation and Discovery

The pro-phages carrying *stx*_2n_ and *stx*_2o_ genes were identified using Phaster (https://phaster.ca, accessed on 11 October 2023). The Stx2 pro-phage region flanked by *attL* and *attR* sites from each genome strain were extracted and annotated using Galaxy tracker Prokka 1.14.6 [25] and visualized with SnapGene Viewer v6 (https://www.snapgene.com/snapgene-viewer, accessed on 11 October 2023).

### 2.9. Data Availability

Raw sequences, along with their limited metadata, are publicly available in the sequence read archive (SRA) housed by the National Center for Biotechnology Information (NCBI) under BioProject PRJNA218110; the accession numbers are shown in Table 2.

## 3. Results

### 3.1. Identification of the Novel Stx2n Subtype

An evaluation of the AMRFinderPlus tool for the detection of stx gene variants in the Pathogen Detection system, found 116 (0.2%) genomes among over 60,000 *E. coli* and *Shigella* genomes screened with stx_2_ B subunit sequences that fell just below the cutoffs for stx_2_ subtypes a through g. Several of the new variants identified were in the process of being characterized and published by other groups (e.g., Stx2j, Stx2m, and Stx2o) [1,2]. Among these 116 genomes, two were positive for a novel Stx2 subtype, designated Stx2n, and the remaining 114 were closely related to recently described subtypes Stx2j [MZ229608 and MZ571121], Stx2o [MZ229604], or Stx2m [OQ054797]. Three strains 2013C-3244 (Stx2n), 2017C-4317 (Stx2n), and 2018C-3367 (Stx2o) were selected for resequencing to generate closed genomes for further analysis.

The in-house stx-subtyping based on whole-genome sequences showed that the stx2 sequences from a representative Stx2n strain shared less than 94.6% nucleic acid sequence identities with other stx2 subtypes. stx2 genes and Stx2 proteins were extracted from the genome assemblies and compared against the GenBank database using NCBI BLAST. These comparisons showed the highest similarity (94.6%) with the Stx2n strain. When comparing sequences of Stx2 holotoxin, Stx2n shared 72.2 to 94.6% similarity with the other 14 described Stx2 subtypes at the nucleic acid level and 83.9 to 95% at the amino acid level (Table 3). The last six amino acids in the A subunit were absent, which is also where the amino acid differences between the seven variants of Stx2j were found. All seven Stx2j variants had identical B subunits. These results suggest that the two provincial STEC strains harbor novel Stx2 subtypes. Based on the standardized nomenclature for Stx2 [4], the new Stx2 subtype was designated Stx2n.

**Figure 1:**
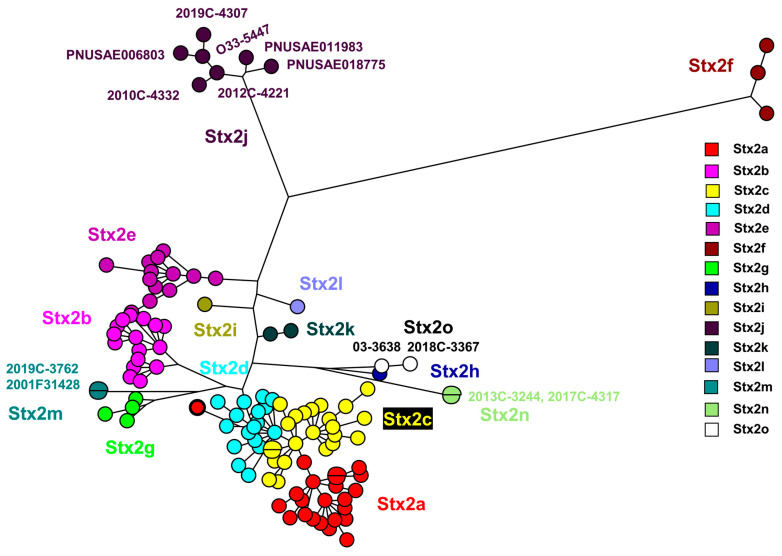
Stx2 subtype amino acid comparison of 117 sequences using maximum parsimony tree. Stx2a, Stx2b, Stx2c, Stx2d, Stx2e, Stx2f, and Stx2g adapted from [4]. Addition of other subtypes adapted from [1,2] Stx2n was added in this study.). The Stx2a outlier (Stx2a-08-BMH-17-0026, Acc. No. MZ229605, circled in black) has an “EDD” motif in the B subunit, and is therefore defined as Stx2a, see Gill et al. [5]. Supplementary Table S2 lists all the sequence information.

**Figure 2:**
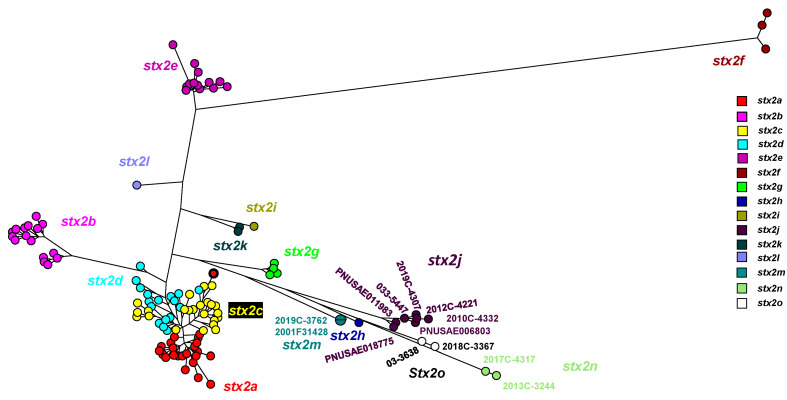
stx2 subtype nucleotide comparison of 117 sequences using maximum parsimony tree. stx2a, stx2b, stx2c, stx2d, stx2e, stx2f, and stx2g adapted from [4]. Addition of other subtypes adapted from [1,2], stx2nwas added in this study. The stx2a outlier (Stx2a-08-BMH-17-0026, Acc. No. MZ229605) in Figure 2 is circled in black. Supplementary Table S2 lists all the sequence information.

The Stx2 subtype amino acid comparison using a maximum parsimony tree (Figure 1) includes the novel strains described here: 2017C-4317, 2013C-3244, 2018C-3367, 03-3638 [5]. Strains 2012C-4221*, 2019C-4307, 2019C-4332, 2010C-4332, PNUSAE011983, and 2019C-3762 from this study, grouped with previously described Stx2j and Stx2m sequences. Supplementary Table S2 list data for all the sequences in Figure 1 and Figure 2.

The Stx2 subtype nucleotide comparison using a maximum parsimony tree (Figure 2) includes the novel strains described in this study: 2017C-4317, 2013C-3244, 2018C-3367, and, previously mentioned, 03-3638 [5]. Strains 2012C-4221, 2019C-4307, 2010C-4332, PNUSAE018775, PNUSAE006803, PNUSAE011983, and 2019C-3762, from this study, were grouped with previously described Stx2j and Stx2m sequences [2]. These strains were included in primer design to ensure the detection of known diversity present in Stx2j and Stx2m.

### 3.2. WGS-Based Characterization of Stx2n and Stx2o Strains

The ten strains in this study are all different serotypes and STs (Table 3) and show variability across time (isolated from 2010 to 2019).

WGS virulence analysis showed that thirty-two virulence genes were harbored between the three isolates. The number of virulence genes varied between the three isolates with 2017C-4317 harboring 9 of 32, 2013C-3244 harboring 18 of 32, and 2018C-3367 harboring 27 of 32 total virulence genes (Table 4). All three isolates harbored *stx2*, *chuA*, *gad*, *kpsE*, *kpsMII_K5*, *sitA*, and *terC* (Table 4). 2017C-4317 harbored virulence genes, *stx2n*, *traT*, and *eilA*. 2013C-3244, harbored additional virulence genes, *focC*, *fyuA*, *iroN*, *irp2*, *iss*, *ompT*, *sfaD*, *sfaS*, *tcpC*, *vat*, and *yfcV*. The virulence profile for 2018C-3367 harbored virulence genes, *stx*2o, *focC*, *fyuA*, *iroN*, *irp2*, *iss*, *ompT*, *sfaD*, *vat*, *yfcV*, *neuC*, *clbB*, *cnf1*, *hra*, *ibeA*, *mchB*, *mchC*, *mchF*, *mcmA*, and *pic* (Table 4). The presence of *fyuA*, *vat*, and y*fcV* qualifies 2013C-3244 and 2018C-3367 as UPEC_HM_ according to the current definition of UPEC [26].

### 3.3. Detection of Shiga Toxin Production

Culture supernatant from each strain was tested for cytotoxicity on Vero cells as described previously [23]. The isolates in this study demonstrated toxicity for Vero cells (Table 5). Additionally, strain 2012C-4221 could be induced when grown with sub-inhibitory concentrations of ciprofloxacin, and toxicity was detected (Table 5). All the strains were positive for *stx*2 when tested with OMNI primers, described in this publication (Table 5). 

### 3.4. Design and Testing of New OMNI PCR Primers

The original stx1 primer to detect all *stx*1 subtypes [4] was redesigned to produce a slightly larger fragment by using a conserved upstream sequence as the new forward primer, now identified as stx1 F3b, and by reversing the original forward primer (stx1-det-F1), now stx1 OMNI-R1 (Table 1). This change allows for the detection of a slightly larger fragment (283 bp) and a clear separation of the *stx*1 fragment from the newly developed fragment for detection of *eae* (221 bp). The *stx*2 primers (stx2-PS8-F, stx2-PS7-R) were redesigned to detect all known *stx*2 subtypes *stx*2a-*stx*2o with a 400bp amplicon, except for *stx*2f. The *stx*2f specific primers are needed for a 438bp amplicon (this study, Table 1). The *eae* primers (PS17 eae-F, PS18 eae-R-NEW) are designed to detect all known *eae* genes; this includes all variants of *eae* found in *Citrobacter* spp. These primers were tested in three different laboratories, using standard reference strains to confirm detection and amplicon size.

### 3.5. Identification of the Stx2n and Stx2o Pro-Phages in the Strains from This Study

**Figure 3:**
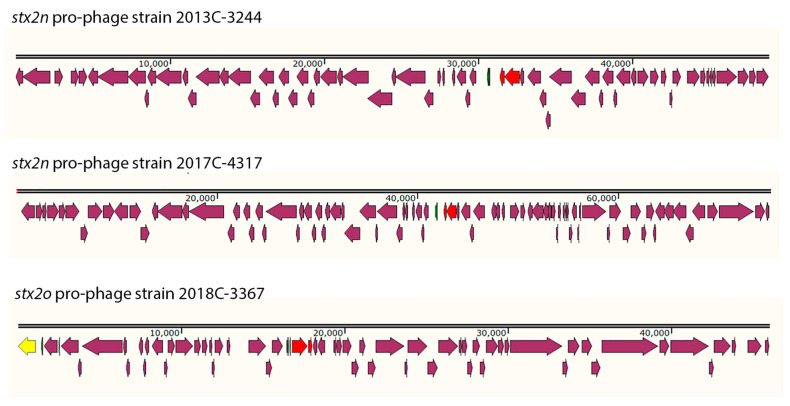
Schematic representation of the Stx2n or Stx2o pro-phages found in the three different strains. The pro-phages are different sizes and not drawn to scale. The stx2 gene A and B units are in red and the integrase is colored yellow.

The Stx2 pro-phages were identified in different locations for each strain from this study (Supplementary Figure S1). The Stx2 pro-phages were all different sizes and gene compositions, even among the two Stx2n strains (Figure 3). Stx2n pro-phage in strain 2013C-3244 was 45.2Kb in size and the %GC content was 49.97. Stx2n pro-phage in strain 2017C-4317 was 75.1Kb in size and the %GC content was 50.76. Stx2o pro-phage in strain 2018C-3367 was 45.9Kb in size and the %GC content was 49.63. The *attL* and *attR* were also different for each Stx2n pro-phage. For strain 2013C-3344, the *attL* sequence for the Stx2n pro-phage was TGGCGAAAAACTG and was located at the following coordinates in the chromosome 2,048,352, while the identical *attR* sequence was located at 2,086,928. For strain 2017C-4317, the *attL* sequence for the Stx2n pro-phage was TTAATTAATTTA and located at the following coordinates in the chromosome 2,907,157, while the identical *attR* sequence was located at 2,982,293. For strain 2018C-3367, the *attL* sequence for the Stx2o pro-phage was TCAATCACTTACA and located at 2,071,326, while the identical *attR* was located at 2,113,169. Most of the genes identified in these three pro-phages coded for hypothetical proteins.

## 4. Discussion

In our present study, Stx-producing STEC strains were isolated from patients in clinical settings in the United States. Six *stx*2j strains were included in Figure 1 and Figure 2 and Table 3 to demonstrate the diversity in the *stx*2j subtype when compared to each other and strains described by Gill et al. [1]. Here, *Stx*2j subtype strains, 2010C-4332, 2012C-4221, 2019C-4307, PNUSAE018775, PNUSAE006803, and PNUSAE011983, were identified in six different serogroups (O158, O162, O32, ONT, O33, and O183) and STs (5662, 5350, 5736, 491, 5923, and 657) over a period of 11 years. The *stx2m* strain, 2019C-3762, was included here to demonstrate the diversity in this subtype when compared to strains described by Bai et al. [2]. The two Stx2n-STEC isolates, 2017C-4317 and 2013C-3244, show diversity in serotype (O23 and O1) and ST (70 and 1385). The Stx2o strain, 2018C-3367, was included here to demonstrate the diversity in this subtype when compared to strains described by Gill et al. [1].

The Stx2 pro-phages for Stx2n and Stx2o strains described here (2013C-3244, 2017C-4317, 2018C-3367) were identified in different locations (Figure 3) and were all different sizes and gene compositions (Table 4), even among the two Stx2n pro-phages (Figure S1). Our findings that the strains carrying these *Stx*2 subtypes have different predicted serogroups, are in separate ST classes, and were isolated in different years and different locations demonstrate that the phages that encode these toxin subtypes are mobile and have spread among different *E. coli*, as has been shown for other *stx*-phages [27].

The virulence gene profile (Table 4) highlights the variability of known virulence genes among the three isolates, with 2017C-4317 harboring 9 of 32, 2013C-3244 harboring 18 of 32, and 2018C-3367 harboring 27 of 32 total virulence genes (Table 4). Of note, the STEC strains, 2013C-3244 and 2018C-3367, carry the genes *fyuA*, *vat*, and *yfcV*, which qualifies these two strains as UPEC_HM_ according to the current definition of UPEC [26]. Strains classified as multiple pathotypes can be more dangerous to human health because once an initial pathotype is detected, analysis may stop, missing additional virulence genes related to a second pathotype related to human illness. The O104 outbreak in Europe was caused by a strain that was both STEC and EAEC [28]. A complete WGS analysis of *E. coli* includes databases from CGE that were designed to provide a complete examination of important genes such as those for serotyping, virulence genes, and pathotype [19,27]. We note that the public contribution of surveillance data by groups such as PulseNet and publicly available analysis results such as MicroBIGG-E (https://www.ncbi.nlm.nih.gov/microbigge, accessed on 11 October 2023) demonstrate the power of large-scale and open data analysis to identify novel genes and variants important to public health.

## Figures and Tables

**Table 1:** Primers for detection of all known Stx1 and Stx2 subtypes. * Wobble bases are shown in bold.

Primer Name	Primer Sequence *	Amplicon Size (bp)	Reference
*stx2*-PS8-F	5′-TCAC**Y**GGTTTCATCATATCTGG	400	This study
*stx2*-PS7-R	5′-GCCTGTC**B**CCA**S**TTATCTGACA		
PS19 *stx2f*-F	5′-GTACAGGGATGCAGATTGGGCG	438	This study
PS20 *stx2f-*R	5′-CTTTAATGGCCGCCCTGTCTCC		
PS17 *eae*-F	5′-CGGCTATTTCCGCATGAGCGG	221	This study
PS18 *eae*-R-NEW	5′AGTT**D**ACACCAAY**W**GTC**R**CCGC		
*stx1* F3b	5′-CTGATGATTGATAGTGGCACAGG	283	This study
*stx1* OMNI-R1	5′-GCGATTTATCTGCATCCCCGTAC		

**Table 2:** Characterization of Stx2j, Stx2m, Stx2n, and Stx2o-carrying STEC strains.

Stx Subtype	CDC Isolate ID	BioSample	ST Type	O:(K):H Type	PNID	Assembly
Stx2j	2010C-4332	SAMN04377066	5662	O158:H23	PNUSAE001889	GCA_012764415.1
Stx2j	2012C-4221	SAMN08579578	5350	O162:H6	None	GCA_003018235.1
Stx2j	2019C-4307	SAMN12361752	5736	O32:K87:H2	PNUSAE027323	GCA_011901845.1
Stx2j	See PNUSAE	SAMN10170522	491	ONT:H45	PNUSAE018775	GCA_003903075.2
Stx2j	See PNUSAE	SAMN07177511	5923	O33:H14	PNUSAE006803	GCA_012463025.1
Stx2j	See PNUSAE	SAMN08595463	657	O183: H18	PNUSAE011983	GCA_012253565.1
Stx2m	2019C-3762	SAMN11569941	9312	O38:H39	PNUSAE024072	GCA_011950125.1
Stx2n *	2013C-3244	SAMN04578435	1385	O1:K22:H4 ^1^	None	GCA_012711215.2 ^2^
Stx2n *	2017C-4317	SAMN07709929	70	O23:H15	PNUSAE009425	GCA_013342905.2 ^2^
Stx2o	2018C-3367	SAMN08799860	80	O75:H7	PNUSAE012694	GCA_012224845.2 ^2^

* Isolates producing the novel stx subtype, Stx2n, described in this study. ^1^ Both lactose positive and negative colonies were found. ^2^ Closed hybrid assemblies generated for this study.

**Table 3:** Nucleotide\amino acid identities (%) using Neighbor Joining comparison in BioNumerics version 8.1 (Applied Maths, Biomérieux), between Stx2n, Stx2o, and representatives of other described Stx2 subtypes. 1. Stx2a (EDL933, X07865), 2. Stx2b (EH250, AF043627), 3. Stx2c (031, L11079), 4. Stx2d (C165-02, DQ059012), 5. Stx2e (S1191 (M21534), 6. Stx2f (F08-101-31, AB472687), 7. Stx2g (7v, AY286000), 8. Stx2h (STEC299, CP022279), 9. Stx2i (CB10366, FN252457), 10. Stx2j (5447, MZ571121), 11. Stx2k (STEC309, CP041435), 12. Stx2l (FHI 1106-1092, AM904726), 13. Stx2m (2001F31428, OQ054797), 14. Stx2n (2017C-4317, GCA_013342905.2), and 15. Stx2o (03-3638, MZ229604). Bold values highlight the sequence identities of identified Stx2n and Stx2o subtypes with previously reported Stx2 subtypes.

Nucleotide\Amino Acid	1	2	3	4	5	6	7	8	9	10	11	12	13	14	15
	*stx*2a	*stx*2b	*stx*2c	*stx*2d	*stx*2e	*stx*2f	*stx*2g	*stx*2h	*stx*2i	*stx*2j	*stx*2k	*stx*2l	*stx*2m	***stx*** **2n**	***stx*** **2o**
1. Stx2a		91.9	98.4	96.9	92.2	70.8	94.2	91.7	93.2	89.5	94.4	95.5	93.2	**87.9**	**89.7**
2. Stx2b	95.3		92.2	93.4	89.4	70.6	91.4	92.2	89.2	89.2	91.3	90.0	90.6	**87.8**	**90.2**
3. Stx2c	99.2	95.4		97.4	91.9	70.5	93.1	91.7	92.4	89.6	94.7	94.8	92.2	**88.1**	**89.7**
4. Stx2d	98.4	96.0	98.5		92.1	70.5	94.0	92.2	92.9	90.2	96.1	94.8	91.5	**88.2**	**90.2**
5. Stx2e	95.2	94.2	94.9	95.7		74.8	92.2	90.2	94.7	88.3	93.8	95.0	88.9	**86.9**	**88.8**
6. Stx2f	81.9	81.2	81.4	82.0	84.1		71.4	71.2	71.3	71.1	71.0	71.5	71.1	**72.2**	**70.4**
7. Stx2g	97.0	95.1	96.5	97.1	95.9	82.2		91.9	94.5	88.2	92.9	92.8	91.2	**87.0**	**89.1**
8. Stx2h	95.2	95.3	95.0	95.6	94.4	81.8	95.5		92.0	92.1	92.8	91.1	92.1	**91.4**	**94.1**
9. Stx2i	95.9	93.7	95.4	96.1	97.3	82.3	96.5	95.4		88.6	96.5	95.1	90.0	**88.1**	**89.4**
10. Stx2j	93.5	93.2	93.7	94.2	92.6	82.7	92.7	93.5	92.5		90.6	89.8	89.2	**88.1**	**90.9**
11. Stx2k	97.2	95.4	97.3	98.2	97.2	82.2	96.8	95.9	97.9	94.1		95.5	90.9	**88.1**	**90.3**
12. Stx2l	96.8	94.1	96.4	97.1	97.3	82.3	96.2	94.7	97.3	93.9	97.8		89.9	**87.4**	**89.9**
13. Stx2m	95.7	95.1	95.3	95.5	94.3	82.0	96.1	94.8	94.5	92.1	95.0	93.8		**87.7**	**88.9**
**14. Stx2n**	**93.2**	**93.1**	**93.4**	**93.6**	**92.6**	**83.9**	**93.5**	**95.0**	**93.1**	**91.8**	**93.9**	**92.4**	**93.2**		**91.4**
**15. Stx2o**	**94.3**	**94.0**	**94.5**	**94.7**	**93.6**	**81.8**	**94.5**	**96.9**	**94.3**	**92.8**	**95.2**	**93.8**	**93.8**	**94.6**	

**Table 4:** Presence of virulence genes in the two Stx2n and Stx2o-producing STEC sequences. A + indicates detection of the gene, a − indicates the gene was not detected.

Virulence Gene	Function	2013C-3244	2017C-4317	2018C-3367
*stx*2n	Shiga toxin 2	+	+	−
*stx*2o	Shiga toxin 2	−	−	+
*chuA*	Outer membrane hemin receptor	+	+	+
*focC*	S fimbrial/F1C minor subunit	+	−	+
*fyuA* *	Siderophore receptor	+	−	*+*
*gad*	Glutamate decarboxylase	+	+	*+*
*iroN*	Enterobactin siderophore receptor protein	+	−	*+*
*irp2*	High molecular weight protein 2 non-ribosomal peptide synthetase	+	−	*+*
*iss*	Increased serum survival	+	−	*+*
*kpsE*	Capsule polysaccharide export inner-membrane protein	+	+	*+*
*kpsMII_K5*	Polysialic acid transport protein; Group 2 capsule	+	+	*+*
*ompT*	Outer membrane protease (protein protease 7)	+	−	*+*
*sfaD*	S fimbrial/F1C minor subunit	+	−	*+*
*sfaS*	S-fimbriae minor subunit	+	−	−
*sitA*	Iron transport protein	+	+	*+*
*tcpC*	Tir domain-containing protein	+	−	−
*terC*	Tellurium ion resistance protein	+	+	*+*
*vat* *	Vacuolating autotransporter toxin	+	−	*+*
*yfcV* *	Fimbrial protein	+	−	*+*
*traT*	Outer membrane protein complement resistance	−	+	*−*
*eilA*	Salmonella HilA homolog	−	+	*−*
*neuC*	Polysialic acid capsule biosynthesis protein	−	−	*+*
*clbB*	Hybrid non-ribosomal peptide/polyketide megasynthase	−	−	*+*
*cnf1*	Cytotoxic necrotizing factor	−	−	*+*
*hra*	Heat-resistant agglutinin	−	−	*+*
*ibeA*	Invasin of brain endothelial cells	−	−	*+*
*mchB*	Microcin H47 part of colicin H	−	−	*+*
*mchC*	MchC protein	−	−	*+*
*mchF*	ABC transporter protein MchF	−	−	*+*
*mcmA*	Microcin M part of colicin H	−	−	*+*
*pic*	serine protease autotransporters of Enterobacteriaceae (SPATE)	−	−	*+*
*usp*	Uropathogenic specific protein	−	−	*+*
Pathotype		STEC/UPEC_HM_	STEC	STEC/UPEC_HM_

* Genes that qualify strains as UPEC_HM_ according to the current definition of UPEC (27).

**Table 5:** Results of Vero Cell Assay.

Stx Subtype	CDC Isolate ID	Log CD_50_/mL Supernatant	PCR Confirmation	Ciprofloxacin Induction
Stx2j	2010C-4332	4.6	+	No
Stx2j	2012C-4221	4.5	+	Yes ^#^
Stx2j	2019C-4307	3.4	+	No
Stx2m	2019C-3762	3.2	+	No
Stx2n	2013C-3244	3.2	+	No
Stx2n	2017C-4317	2.0 ^	+	No
Stx2o	2018C-3367	3.0	+	No

^#^ Toxin is detectable after the strain is grown in the presence of ciprofloxacin, ^ cell-associated toxicity.

## Data Availability

The whole genome sequence data for this study are publicly available at NCBI. Specific NCBI accession numbers are listed in Table 3 and in Supplementary Tables S1 and S2. The findings and conclusions in this report are those of the author(s) and do not reflect the view of the Centers for Disease Control and Prevention, the Department of Health and Human Services, or the United States government. Furthermore, the use of any product names, trade names, images, or commercial sources is for identification purposes only, and does not imply endorsement or government sanction by the U.S. Department of Health and Human Services.

## Supplementary Materials

The following supporting information can be downloaded at https://www.mdpi.com/article/10.3390/microorganisms11102561/s1,. Table S1: Statens Serum Institut strain collection of reference strains harboring the *stx* gene subtypes, their O:H serotype, additional virulence genes, and identification numbers [1,2,5,6,29,30,31,32,33,34,35,36,37,38,39]. Table S2: Sequence information including accession numbers, protein, and nucleotide sequences. Figure S1: Location of pro-phages identified by Phaster (https://phaster.ca) in the chromosome of the strains carrying novel Stx2 types (Stx2n pro-phage and Stx2o pro-phage) reported in this study [40].
