# Supplementary material for: Identification and Characterization of ten Escherichia coli Strains Encoding Novel Shiga Toxin 2 Subtypes, Stx2n as Well as Stx2j, Stx2m, and Stx2o, in the United States
Source: Microorganisms. 2023 Oct 14;11(10):2561. doi: 10.3390/microorganisms11102561 (PMC10608928; doi:10.3390/microorganisms11102561)

Supplementary Materials:

Supplementary Table S1. Statens Serum Institut strain collection of reference strains harboring the *stx* gene subtypes, their O:H serotype, additional virulence genes, references and identification numbers.

SSI collection D number	Strain [referenced in main manuscript]	Control for toxin subtype	Toxin variant designation	GenBank accession No.	Results obtained using the subtyping method in [4]	Serotype	Additional virulence genes
D2653 [1]	EDL933 [29]	Stx1a	Stx1a-O157-EDL933	M19473	*stx1a + stx2a*	O157:H7	*eae, ehxA, astA*
D3602	DG131/3 [30,31]	Stx1c	Stx1c-O174-DG131-3	Z36901	*stx1c + stx2b*	O174:K101:H8	
D3522	MHI813 [32]	Stx1d	Stx1d-O8-MHI813	AY170851	*stx1d*	O8:K85ab:Hrough	
D2653[1]	EDL933 [29]	Stx2a	Stx2a-O157-EDL933	X07865	*stx1a + stx2a*	O157:H7	*eae, ehxA, astA*
D3428	EH250 [33]	Stx2b	Stx2b-O118-EH250	AF043627	*stx2b*	O118:K?:H12	*astA*
D2587 [2]	031 [34]	Stx2c	Stx2c-O174-031	L11079	*stx2c + stx2b*	O174:K101:H21	
D3435 [3]	C165-02 [35]	Stx2d	Stx2d-O73-C165-02	DQ059012	*stx2d*	O73:H18	*astA*
D3648	S1191 [36,39]	Stx2e	Stx2e-O139-S1191	M21534	*stx2e*	O139:K12:H1	
D3546	T4/97 [37]	Stx2f	Stx2f-O128-T4-97	AJ010730	*stx2f*	O128ac:[H2]	*eae, bfpA, astA*
D3509	7v [38]	Stx2g	Stx2g-O2-7v	AY286000	*stx2g*	O2:K-:H25	*ehxA, astA, estap*
D6061	STEC299 [5]	Stx2h	Stx2h-O102-STEC299	CP022279	*stx2*	O102:K+:H18	*paa*, *kpsM*II_K5
D6205A	CB10366 [6]	Stx2i	Stx2i-O9-CB10366	FN252457	*stx2*	O9:K39:[H4]	*estb*
D6283	OLC-1685 [1]	Stx2j	Stx2j-O158-OLC-1685	MZ229608	*stx2*	O158:H23	
D6068	12GZSW01 [39]	Stx2k	Stx2k-O159-12GZSW01	KC339670	*stx2*	O159:K?:H16	*astA*, *estap*, *estb*
D7138	1610T27873 [6]	Stx2l	Stx2l-O65-1610T27873	AM904726 [4]	*stx2a + stx2d*	O65:H16	*hlyE*, *lpfA*, *shiB*, *tia*
D6276	2001F31428 [2]	Stx2m	Stx2m-O96-2001F31428	OQ054797	*stx2*	O96:H19	
D6924	2017C-4317[5]	Stx2n	Stx2n-O23-2017C-4317	CP113092	*stx2*	O23:K-:H15	*chuA*, *kpsE*, *eilA*, *traT*, *kpsMII_K5*, *sitA*,

D6282	03-3638 [1]	Stx2o	Stx2o-O85-03-3638	MZ229604	*stx2*	O85:H1	

[1] This strain is used for both Stx1a and Stx2a
[2] This strain has been replaced by D3431, serotype O157:H7, *stx2c, eae, ehxA, astA*
[3] May result in both fragments at 179 bp and 280 bp; This strain has been replaced by D4134, serotype O166:H15, *stx2d*; ESBL producing.
[4] This is the original Acc. No. for strain FHI 1106-1092 of serotype O8:H2. The presence of the *stx2l* gene is however very unstable and a similar Danish isolate is used as the reference strain for Stx2l.
[5] Stx2n strain is from this study.

Supplementary Figure S1. Location of pro-phages identified by Phaster (https://phaster.ca) in the chromosome of the strains carrying novel stx2 types (stx2n and stx2o) reported in this study [40]. The stx2 pro-phages regions are highlighted for each strain.

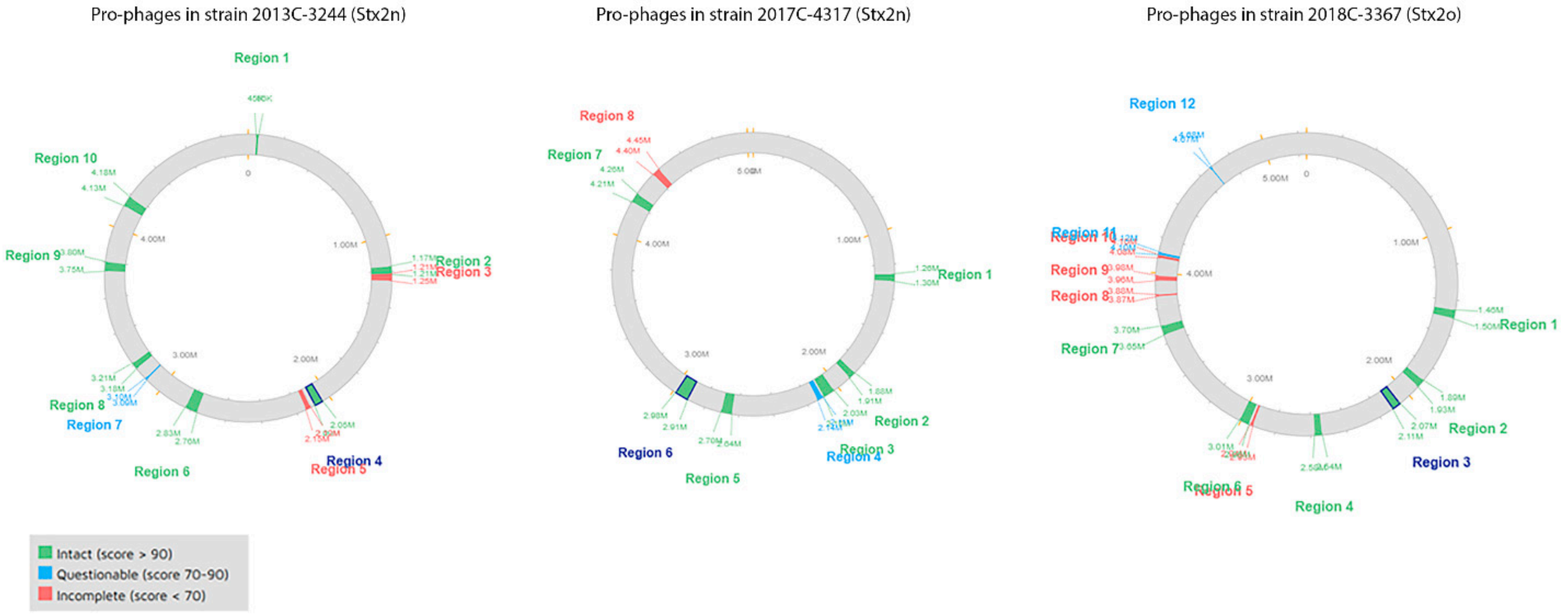

Supplement: Supplementary file 1 [file microorganisms-11-02561-s001.zip › RLindsey_Supplementary_Materials.pdf]
